# Low-cost, low-power, clockwork syringe pump

**DOI:** 10.1016/j.ohx.2023.e00469

**Published:** 2023-08-30

**Authors:** Francis Pooke, Matthew Payne, Lui Holder-Pearson, Doug Heaton, Jake Campbell, J. Geoffrey Chase

**Affiliations:** Department of Mechanical Engineering, University of Canterbury, New Zealand

**Keywords:** Escapement, Spring-driven, Programmable, Micro-litre pump, Insulin pump

## Abstract

A low-cost ($120 NZD, $75 USD), low-power (1-year battery life), portable, and programmable syringe pump design is presented, which offers an alternative to high-cost commercial devices with limited battery life. Contrary to typical motor-driven syringe pumps, the design utilizes a compression spring coupled with a clockwork escapement mechanism to advance the syringe plunger. Full control over flow-rate and discrete (bolus) deliveries is achieved through actuation of a clockwork escapement using programmable, low-power electronics. The escapement mechanism allows the syringe plunger to advance a fixed linear distance, delivering a dose size of 0.001 ml in the configuration presented. The modular pump assembly is easily reconfigured for different applications by interchanging components to alter the minimum dose size. Testing to IEC 60601-2-24(2012), the average error of the clockwork syringe pump was 8.0%, 4.0%, and 1.9% for 0.001 ml, 0.002 ml, and 0.01 ml volumes, respectively. An overall mean error of 1.0% was recorded for a flow-rate of 0.01 ml h^−1^. Compared to a commercial insulin pump, the clockwork pump demonstrated reduced variability but greater average error due to consistent over-delivery. Further development of the design and/or manufacture should yield a device with similar performance to a commercial pump.

## Graphical abstract

**Figure:**
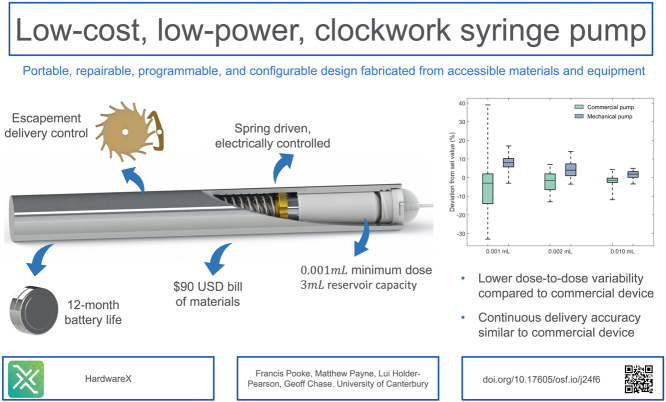

## belowfloat

NZD: New Zealand Dollar
USD: United States Dollar
IEC: International Electrotechnical Commission
ISO: International Organization for Standardization
ANSI: American National Standards Institute
AGMA: American Gear Manufacturers Association
CAD: Computer-Aided Design
EDM: Electrical Discharge Machining
PCB: Printed Circuit Board
SPI: Serial Peripheral Interface
ID: Internal Diameter
OD: Outer Diameter
CSII: Continuous Subcutaneous Insulin Infusion

**Table:** 

Specifications table	

Hardware name	*Low-cost, low-power, clockwork syringe pump*

Subject area	•Mechanical Engineering • Biomedical Engineering • Medical devices

Hardware type	•Mechanically-driven syringe pump • Low-cost device for controlled insulin delivery

Closest commercial analog	•Syringe pump • Insulin pump

Open source license	Creative Commons Attribution - ShareAlike 4.0 International (CC BY-SA 4.0)

Cost of hardware	≈$120 NZD ($75 USD)

Source file repository	https://doi.org/10.17605/osf.io/j24f6

### 1 Hardware in context

Syringe pumps control the movement of fluid by mechanically advancing (or retracting) a syringe plunger. Large, externally-powered syringe pumps are commonly used in laboratory applications, and for delivering medication in acute and intensive care scenarios. Devices for medication delivery require very small delivery volumes with low variability. Extension of technologies with small delivery capability to portable applications is hampered by high power consumption, or prohibitive expense. For example, syringe pumps are used for continuous subcutaneous insulin infusion (CSII) in diabetes outpatient care via insulin pumps [1], [2], [3], [4]. Insulin pumps present an extreme case for portable syringe pump devices in terms of size, while also requiring small, precise volumes to be delivered.

A significant limitation of syringe pumps is cost. In New Zealand, insulin pumps cost between $7000–10,000 NZD ($4400–$6250 USD). In addition to large upfront cost, they are not typically able to be repaired or serviced. For relatively simple hardware – a motor-driven leadscrew which advances a plunger – this cost is very high. For insulin pumps in particular, pump cost prevents more universal uptake of CSII, which is regarded the gold-standard for glucose control and diabetes outpatient care [5].

One further common problem with motor-driven syringe pumps is a large electrical energy requirement, as all energy to deliver fluid must be provided by an electrical power source. For portable devices, this energy requirement results in either large, inconvenient batteries, or limited battery life. Commercially available insulin pumps, for example, require batteries to be changed approximately every 1–4 weeks.

The device presented here provides a portable, low-cost, low-power, and programmable syringe pump. It achieves great generalizability through configurable components, but is intended for eventual use as an insulin pump. The design operates using a spring-driven clockwork mechanism with electrical actuation, which lends itself to long battery life, as electrical energy is only used for dose control, rather than to advance the plunger. Flow-rate and bolus dose functionality are provided, and can be modified via program. Cost is minimized through selection of off-the-shelf components for a total bill of materials of $120 NZD ($75 USD), depending on access to mechanical and electronic workshops. The modular design enables users to easily perform repairs or modifications, increasing potential application in low-resource regions.

There are currently many open-source syringe pump designs suitable for a wide-range of research applications. These designs address issues of commercial pumps, including cost, compatibility, and lack of customizability. The design developed by [6] offers similar performance to a commercial syringe pump for ≈5% of the cost. It fits any size syringe, and is controlled using a web-interface. Similar open-source devices include a design targeting fluid delivery for behavioural neuroscience [7], a touch-screen controlled device for life science applications [8], a dual-syringe device for chemistry lab use [9], and a generic syringe pump constructed predominately by re-purposing an open-source 3D printer [10]. While inexpensive, easy to build, and customizable for different lab-based research applications, the designs all operate using a motor-driven leadscrew to advance a syringe plunger, and are powered using an external power source. None change the drive mechanism to meet other criteria. Therefore, the devices are not appropriate for field-based use, insulin therapy, or other applications where a highly portable, self-powered device is required.

An open-source insulin pump design developed by [11] is portable, and powered by an AAA battery. However, the design also operates using a motor-driven leadscrew, which limits battery life to the ≈1–4 weeks of commercial insulin pumps. The clockwork design presented here is differentiated from other open-sourced designs by its unique spring-driven, escapement-controlled actuation mechanism. It will enable a significantly longer, ≈1 year battery life (estimated). However, compared to this open-source insulin pump, the presented configuration of the clockwork pump cannot achieve the same delivery resolution, with a minimum dose size of 0.001 ml, compared to 0.25 × 10^-3^ ml (4× smaller) achieved by the open-source insulin pump [12].

Portable and mechanical pump designs can also be found in literature, including a device for microfluidic applications, which utilizes a compression spring to drive fluid stored in a cartridge [13]. Flow control is achieved using passive valves in a microfluidic chip. Another pump targeted at portable microfluidics advances the syringe plunger using a rack and pinion configuration, powered by a helical spring [14]. Flow-rate is controlled using a clockwork escapement actuated by a balance spring. Both designs operate without electronics (no battery or external power required). As a result, these devices are highly portable, reliable, and well-suited for continuous-flow applications. However, as dose timing is not controlled electrically, these pumps cannot be programmed for different flow-rates, or deliver discrete doses requested by the user, rendering them incompatible with insulin therapy or many other applications requiring variable dosing over time. In contrast to other open-source syringe pumps, the design presented here offers a small, portable, low-cost, and low-power device, which can be programmed for different flow-rates and/or discrete deliveries.

### 2 Hardware description

**Fig. 1:**
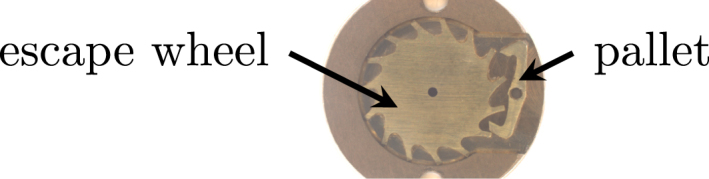
Escape wheel and pallet used for dosage control in the clockwork pump.

The clockwork pump is a modular, cylindrical syringe pump, which displaces fluid from a reservoir using a spring-driven plunger. The plunger is attached to a threaded nut, which back-drives a leadscrew, inducing rotation. Dose volume is controlled using a clockwork escapement mechanism which prevents uncontrolled release of the gear train [15]. The escapement consists of an oscillating component (pallet) and a club-toothed gear (escape wheel), as shown in Fig. 1. The escape wheel is connected through a 60:1 gearbox to the leadscrew. Free rotation of the escape wheel is interrupted by the pallet, which is coupled to a low-power actuator with an inbuilt spring-return mechanism. Each activation of the actuator allows the escape wheel to rotate 1/15th of a revolution, enabling the plunger to advance 9 µm, displacing a 0.001 ml discrete dose from the reservoir.

As the force provided by the spring to the plunger decreases as the plunger advances (as the spring decompresses), the achievable pumping pressure linearly decreases from peak pressure at the fully retracted plunger position (maximum spring compression) to minimum pressure at the fully extended plunger position (minimum spring compression). For the presented design, the pressure range is 175 kPa to 50 kPa, which is considered suitable for initial testing and development of a device targeting insulin therapy. However, the achievable pressure range can easily be altered for a different pumping application by varying the stiffness of the spring. The method used to select a spring with adequate stiffness for insulin therapy is outlined in Section 7.4, and can be adapted for any pumping application with a given minimum (or maximum) pressure requirement.

Provided the required pumping pressure for a given application can be met by the minimum achievable pressure of the pump, the dose delivered each actuation across the entire working range of the plunger will remain constant at 0.001 ml. A constant dose size is achieved as the escapement mechanism restrains the lead-screw to a fixed rotation each actuation, so greater spring force does not result in larger lead-screw rotations or subsequent linear plunger movement. Therefore, pump accuracy is independent from plunger position, provided the spring force can generate sufficient pumping pressure.

As the pump is intended for eventual use as an insulin pump, the device must be capable of providing both pseudo-continuous flow-rates, and one-off ‘bolus’ doses. In insulin therapy, the continuous flow-rate provides a background level of insulin to manage blood sugar levels between meals, and boluses are additional doses programmed by a user to compensate for meals, or to correct blood sugar levels. The presented pump design can provide modifiable flow-rates, and deliver bolus doses when connected to an external controller. The current device does not have Bluetooth connectivity or a built-in button, so a user cannot directly program a bolus dose.

Flow-rates are achieved by repeated activations of the low-power actuator at set intervals, controlled by an on-board micro-controller. Flow-rate is governed by the interval between each 0.001 ml dose (cycle time), which can be modified by program. The minimum achievable flow-rate is a function of the per-actuation dose volume, and the maximum acceptable cycle time for a certain application. If a cycle time of 1 h is considered acceptable, the minimum hourly flow-rate is 0.001 ml h^−1^. The maximum flow-rate is governed by the minimum possible cycle time (approximately 100 ms), allowing a flow-rate of 36 ml h^−1^. The practicality of the maximum flow-rate is limited in the current design by the reservoir volume of 3 ml, allowing the flow-rate to be sustained for only 5 min before the reservoir is depleted. With all flow-rates comprised of the same 0.001 ml individual increments, flow-rate is unaffected by plunger position. Pump accuracy is also not impacted by flow-rate.

Bolus deliveries of various size are possible through repeated activations of the actuator at a cycle time of 100 ms, providing rapid delivery. The minimum possible bolus size is 0.001 ml. The maximum possible bolus size depends on the period of time the dose must be delivered in. If an arbitrary period of 10 s is adopted, a maximum bolus size of 0.1 ml could be requested. Flow-rate and bolus accuracy are both tested to IEC standard in Section 7.1.

**Table 1:** List of design files.

Design filename	File type	Open source license	File location
Leadscrew_Housing	SLDPRT	CC BY-SA 4.0	physical/
Gearbox_Connector	SLDPRT	CC BY-SA 4.0	physical/
Gearbox_Housing	SLDPRT	CC BY-SA 4.0	physical/
Escapement_Support	SLDPRT	CC BY-SA 4.0	physical/
Escape_Wheel	SLDPRT	CC BY-SA 4.0	physical/
Escape_Wheel	DXF	CC BY-SA 4.0	physical/
Pallet	SLDPRT	CC BY-SA 4.0	physical/
Pallet	DXF	CC BY-SA 4.0	physical/
Actuator_Support	SLDPRT	CC BY-SA 4.0	physical/
Pallet_Lever	SLDPRT	CC BY-SA 4.0	physical/
Pallet_Lever	DXF	CC BY-SA 4.0	physical/
Motor_Mount	SLDPRT	CC BY-SA 4.0	physical/
Electronic_Housing	SLDPRT	CC BY-SA 4.0	physical/
Plunger	SLDPRT	CC BY-SA 4.0	physical/
Reservoir_Housing	SLDPRT	CC BY-SA 4.0	physical/
Spring_Housing	SLDPRT	CC BY-SA 4.0	physical/
b1	KiCAD project	CC BY-SA 4.0	electrical and software/
b2	KiCAD project	CC BY-SA 4.0	electrical and software/
continuousDelivery	Arduino	CC BY-SA 4.0	electrical and software/

There is a significant difference between this design and current commercial, and open-source syringe pump designs. Other designs typically rotate a leadscrew (and advance a plunger) using a motor. The force required to deliver the fluid must be provided solely by the motor, which results in a greater electrical power requirement compared to this clockwork design. It is estimated the novel spring-based drive mechanism will offer a battery life of approximately 1 year, powered by two CR1632 cells, offering >10× improvement over current devices. In addition, the pump can be built for $120 NZD ($75 USD), which represents a 60–80× cost reduction compared to a commercial insulin pump. The current prototype is lightweight and portable, weighing 106 g, and measuring 20 mm in diameter and 120 mm in length.

The clockwork pump is also highly configurable for different applications. The leadscrew, gearbox, and reservoir can be changed to alter the pumping resolution. The design is modular, consisting of 6 separate sub-assemblies, which can be replaced or redesigned without impacting other sub-assemblies. Although the design is well-suited for medical applications, it is a prototype, and does not meet the safety requirements required for medical device certification. The pumping mechanism and design analysis presented will be useful to researchers who wish to further develop the concept for a wide variety of applications.

The clockwork pump:

• Enables manufacture of a syringe pump for $120 NZD ($75 USD), with battery life of ≈ 1 year;
• Provides a highly customizable design;
• Allows for easy and inexpensive repair, and continued development; and
• Provides researchers with a novel pumping concept able to be developed for medical applications, including insulin therapy.

### 3 Design file summary

The design files are all available at https://doi.org/10.17605/osf.io/j24f6. The file locations specified in Table 1 are relative to the base of this directory.

#### 3.1 CAD files

Physical design was completed in SolidWorks. CAD files for all components to manufacture are provided as SolidWorks Part files (SLDPRT). Component drawings for manufactured components are included in https://doi.org/10.17605/osf.io/j24f6 located under component drawings. DXF files for the escape wheel, pallet, and pallet lever are included in place of drawings, as these components are wire-cut.

#### 3.2 Electronics and software

PCB design was performed in KiCAD. Local library files are included for both the schematics and PCB footprints. Production files for PCB B1 and PCB B2 are provided in b1.zip and b2.zip, respectively. There is minimal software included as only the pump hardware is presented here. An Arduino script providing slow, continuous administration – continuousDelivery.ino – is included only, but can be expanded for any type of basic control [16].

### 4 Bill of materials

Manufactured and purchased components are listed in Table 2, Table 3 respectively. The total device cost is $123 NZD ($75 USD). A detailed bill of materials with all components, and relevant details is provided in https://doi.org/10.17605/osf.io/j24f6. All subsequent references to component numbers are consistent with the identification numbers in Table 2, Table 3.

**Table 2:** Components to manufacture, with all costs in NZD.

No.	Part	Assembly	Material	Cost(NZD)
1	Leadscrew housing	Leadscrew	Aluminium round bar	$2.21
2	Gearbox connector	Gearbox	Aluminium round bar	$2.21
3	Gearbox housing	Gearbox	Aluminium round bar	$2.21
4	Escapement support	Escapement	Brass round bar	$0.80
5	Escape wheel	Escapement	Brass sheet	$2.82
6	Pallet	Escapement	Brass sheet	$2.82
7	Actuator support	Escapement	Brass round bar	$0.80
8	Pallet lever	Escapement	Brass sheet	$2.82
9	Motor mount	Actuator	Aluminium round bar	$2.21
10	Electronics housing	Actuator	Aluminium round bar	$2.21
11	Plunger	Reservoir	Aluminium round bar	$2.21
12	Reservoir housing	Reservoir	Aluminium round bar	$2.21
13	Spring housing	Reservoir	Aluminium round bar	$2.21

Total				$27.74

**Table 3:** Components to purchase, with all costs in NZD.

No.	Part	Assembly	Number	Unit cost (NZD)	Total cost(NZD)
14	Ball bearing	Leadscrew	1	$2.30	$2.30
15	Thrust bearing	Leadscrew	1	$3.20	$3.20
16	Leadscrew	Leadscrew	1	$4.95	$4.95
17	Circlip	Leadscrew	1	$0.30	$0.30
18	One-way bearing	Gearbox	1	$4.50	$4.50
19	Gearbox	Gearbox	1	$15.50	$15.50
20	Pallet shaft	Escapement	1	$1.50	$1.50
21	Miniature shutter motor	Actuator	1	$4.60	$4.60
22	M1.6 × 16 mm socket screw	Actuator	2	$0.16	$0.32
23	M1.6 × 5.5 mm spacer	Actuator	2	$2.23	$4.45
24	M1.6 × 14 mm socket screw	Electronics	2	$0.18	$0.36
25	M1.6 × 10 mm spacer	Electronics	2	$2.23	$4.45
26	PCB B1	Electronics	1	$0.65	$0.65
27	PCB B2	Electronics	1	$0.65	$0.65
28	2 Pos cable	Electronics	1	$1.22	$1.22
39	3 Pos cable	Electronics	1	$1.26	$1.26
30	CR1632 battery	Electronics	2	$3.02	$6.04
NA	PCB components	Electronics	1	$10.49	$10.49
31	Leadscrew nut	Reservoir	1	$1.95	$1.95
32	3 mlreservoir	Reservoir	1	$5.90	$5.90
33	Infusion set	Reservoir	1	$15.30	$15.30
34	Compression spring	Reservoir	1	$2.50	$2.50
35	M2.5 × 3 mm grub screw	Multiple	6	$0.55	$3.30

Total					$95.69

### 5 Build instructions

#### 5.1 Overview of build process

**Fig. 2:**
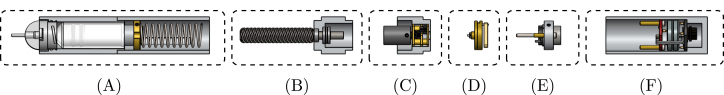
Breakdown of the 6 sub-assemblies in the clockwork pump.

Four key steps are required for the manufacture and assembly of the pump: manufacture of machined components; modification of purchased components; population of PCBs; and construction and integration of sub-assemblies. The six pump sub-assemblies are shown in Fig. 2, and identified in Table 4. The tools and equipment required for all manufacture, modification, and assembly tasks are listed in Table 5.

**Table 4:** Component list for 6 key pump sub-assemblies.

Identifier	Sub-assembly	Components
A	Reservoir	11, 12, 13, 31, 32, 33, 34
B	Leadscrew	1, 14, 15, 16, 17
C	Gearbox	2, 3, 18, 19, 35
D	Escapement	4, 5, 6, 7, 8, 19, 20
E	Actuator	9, 21, 22, 23, 35
F	Electronics	10, 24, 25, 26, 27, 28, 29, 30

**Table 5:** Tools and equipment required for component manufacture, modification, and assembly.

Tools/equipment	Required for
Mill, lathe	Manufacture and modification of multiple components
Tap/die	Manufacture and modification of multiple components
Broaching tool	Manufacture of spring housing
Wire EDM	Manufacture of escape wheel, pallet, and pallet lever
Saw	Modification of pinion and pallet shafts
Scalpel	Modification of shutter motor
Screwdrivers	Modification of gearbox
Soldering iron	Modification of shutter motor
Vice/press	Assembly of leadscrew and gearbox sub-assemblies
Pliers	Assembly of leadscrew sub-assembly
Hex keys	Assembly of gearbox, actuator, electronics, and reservoir sub-assemblies
Kapton tape	Assembly of electronics sub-assembly
SMT P & P machine	Assembly of PCB B1 and PCB B2
Loctite	Assembly of escapement and reservoir sub-assemblies

#### 5.2 Manufacture of machined components

Manufacture components in Table 2 using the component drawings. The escape wheel, pallet, and pallet lever require wire EDM for manufacture. DXF files for these components are provided in https://doi.org/10.17605/osf.io/j24f6. Required tooling and equipment are listed in Table 5. For ease of manufacture, multiple components can be machined from the same stock. Table 6 provides material dimensions enabling all components to be fabricated. Individual part dimensions and possible material suppliers are provided in the detailed bill of materials.

**Table 6:** Pre-manufacture material dimensions for fabrication of all pump components.

Material	Dimensions	Components
Aluminium round bar	20 mmOD × 300 mm	1, 2, 3, 9, 10, 11, 12, 13
Brass round bar	16 mmOD × 30 mm	4, 7
Brass sheet	30 mm × 30 mm × 1.2 mm	5, 6, 8

#### 5.3 Population of PCBs

Populate the PCBs in accordance with the KiCad project files. Table 7 specifies the PCB components required. More detail, including supplier links, is provided in the detailed bill of materials, including non-essential components. A solder paste stencil and a pick and place machine – manual or automatic – greatly assist the PCB population process.

**Table 7:** PCB component list.

Component	Board	Number	Unit cost (NZD)	Total cost (NZD)
Contact spring	B1, B2	7	$ 0.50	$3.50
2 Pos connector header	B1	1	$ 0.48	$0.48
3 Pos connector header	B1, B2	2	$ 0.69	$1.38
10 kΩresistor	B1	1	$ 0.27	$0.27
8 VN-Channel mosfet	B1	1	$ 0.53	$0.53
Linear voltage regulator	B2	1	$ 1.15	$1.15
0.1 µFcapacitor	B2	3	$ 0.10	$0.30
ATTiny 441 IC MCU	B2	1	$ 1.36	$1.36
6 Pos connector header	B2	1	$ 0.69	$0.69

#### 5.4 Modification of off-the-shelf components

**Fig. 3:**
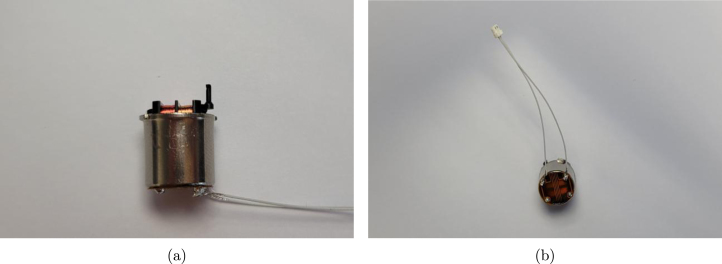
(a) Removal of actuator arm and hook (b) Removal of flex circuit and connection of 2 position cable.

Instructions for modifying the purchased components are listed in Table 8. The two modification steps for the shutter motor (using components 21 and 28) are shown separately in Fig. 3, Fig. 3.

#### 5.5 Assembly

**Table 8:** Modification instructions. Component numbering per Table 2, Table 3.

Component(s)	Modification
16	Use the leadscrew modification drawing to turn a shoulder and groove onto the leadscrew shaft, and cut to length.
19 + 20	Cut the pallet shaft and gearbox pinion shaft down to 10 mm .
21 + 28	
	a Using a scalpel, remove one of the two actuator lever arms. Remove the hook at the end of the remaining arm.
	b Using a scalpel, cut the flex circuit from the shutter motor, trimming the edges so they do not extend out from the motor body. Cut the connections off one end of the 2 position cable, and solder to the exposed wires of the 460 Ω coil.
23	Cut two 5.5 mm sections from the brass tube. Ensure the exposed ends are flat.
25	Cut two 10 mm sections from the brass tube. Ensure the exposed ends are flat.
31	Use the leadscrew nut modification drawing to turn down the flange of the nut, and drill and tap two holes for grub screws.

##### 5.5.1 Leadscrew sub-assembly

**Fig. 4:**
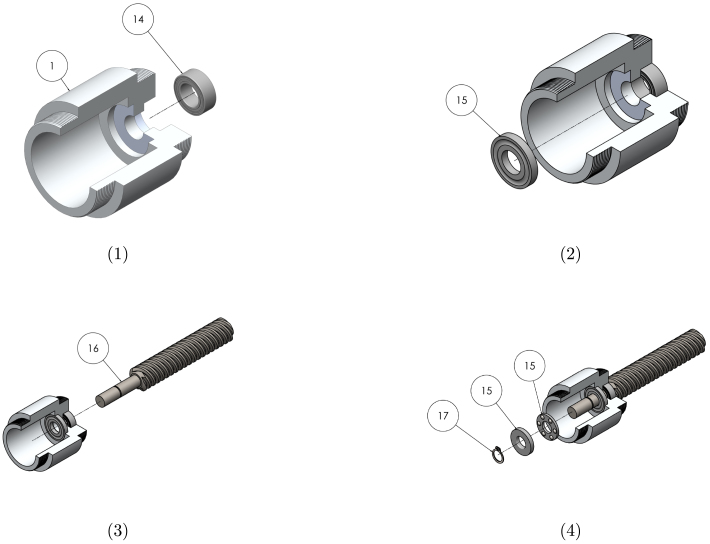
Graphical build instructions for the leadscrew sub-assembly.

Graphical instructions for assembling the leadscrew sub-assembly (sub-assembly B as identified in Fig. 2) are provided in Fig. 4. Each assembly step is detailed in Table 9. Components are numbered with reference to Table 2, Table 3.

##### 5.5.2 Gearbox sub-assembly

**Fig. 5:**
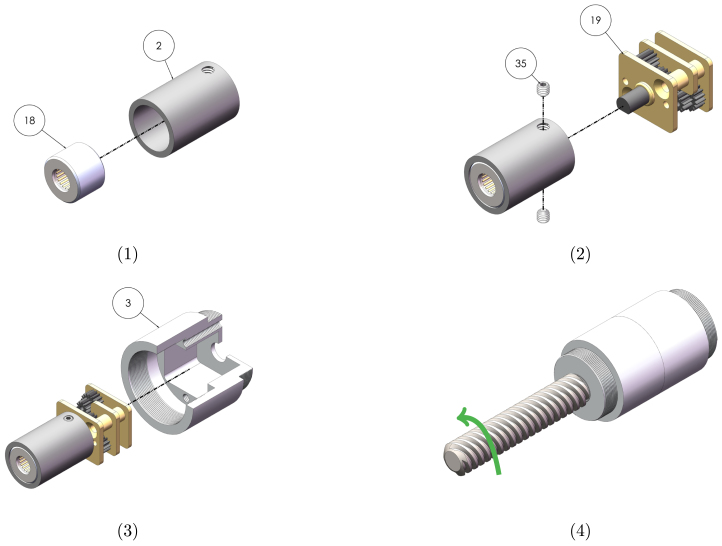
Graphical build and integration instructions for the gearbox sub-assembly.

Graphical instructions for assembling and integrating the gearbox sub-assembly (sub-assembly C as identified in Fig. 2) are provided in Fig. 5. Each assembly step is detailed in Table 10. Components are numbered with reference to Table 2, Table 3.

**Table 9:** Assembly instructions for the leadscrew sub-assembly. Component numbering per Tables 2–3.

Step	Instruction
1	Press the ball bearing (14) into the leadscrew housing (1) using a vice or press.
2	Press the clearance washer (larger ID) of the thrust bearing (15) into the housing using a vice or press. A piece of rod or tube will be required to press on the surface of the washer. Ensure the washer is orientated so the grooves are exposed.
3	Insert the leadscrew (16) into the housing so that ball bearing sits against the shoulder of the leadscrew.
4	Insert the ball cage onto the leadscrew shaft, followed by the remaining thrust washer (grooves facing inwards). Using pliers, place the circlip (17) onto the shaft. Push the circlip down the shaft using spare tube to seat it in the shaft groove.

##### 5.5.3 Escapement sub-assembly

**Fig. 6:**
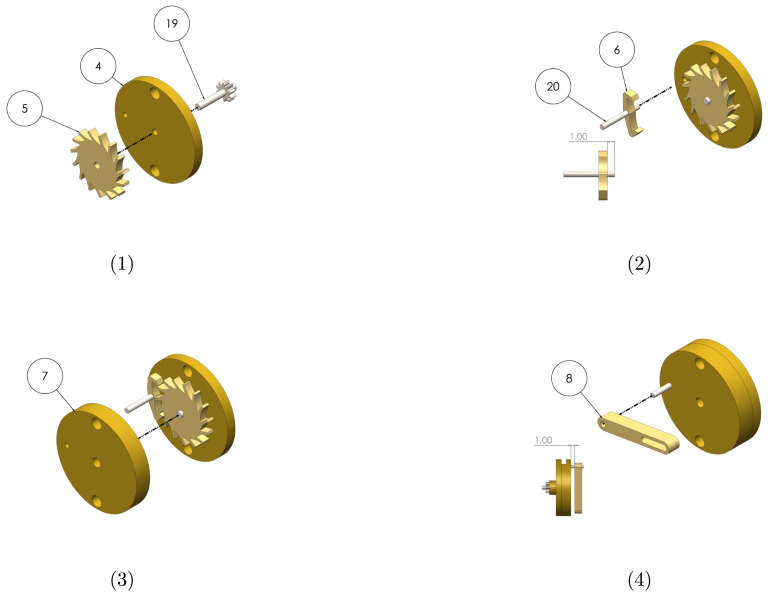
Graphical build instructions for the escapement sub-assembly.

Graphical instructions for assembling the escapement sub-assembly (sub-assembly D as identified in Fig. 2) are provided in Fig. 6. Each assembly step is detailed in Table 11. Components are numbered with reference to Table 2, Table 3.

**Table 10:** Assembly and integration instructions for the gearbox sub-assembly. Component numbering per Tables 2–3.

Step	Instruction
1	Press the one-way bearing (18) into the leadscrew-gearbox connector (2) using a vice or press. Ensure the bearing is orientated so the leadscrew can rotate freely in the direction indicated in step 4 of Fig. 5, but lock in the opposite direction.
2	Insert the shaft of the gearbox (19) into the connector so that the connector sits up against the flange of the gearbox. Insert grub screws (35) into the threaded holes, and tighten onto the shaft using a 1.5 mm hex key.
3	Press the gearbox and connector into the gearbox housing (3) so that back plate of the gearbox sits on the bottom face of the rectangular cut-out.
4	Integrate the leadscrew and gearbox sub-assemblies by pushing the one-way bearing onto the leadscrew shaft, and screwing the housing components together.

##### 5.5.4 Actuator sub-assembly

**Fig. 7:**
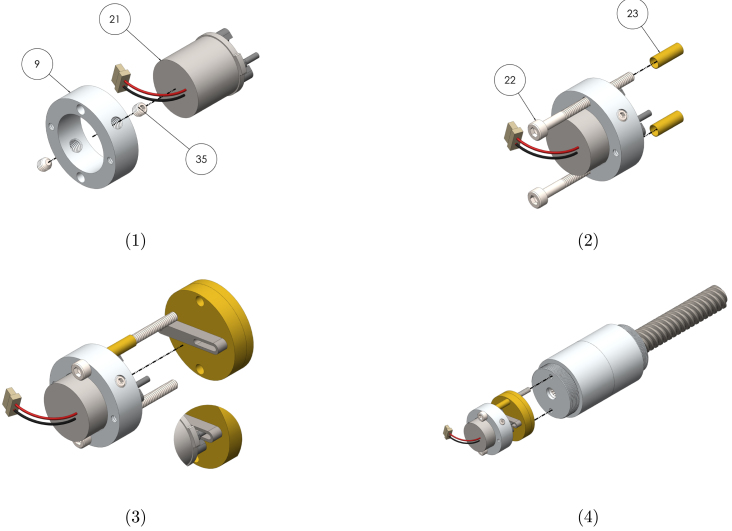
Graphical build and integration instructions for the actuator sub-assembly.

Graphical instructions for assembling and integrating the actuator sub-assembly (sub-assembly E as identified in Fig. 2) are provided in Fig. 7. Each assembly step is detailed in Table 12. Components are numbered with reference to Table 2, Table 3.

**Table 11:** Assembly instructions for the escapement sub-assembly. Component numbering per Tables 2–3.

Step	Instruction
1	Insert the pinion shaft (19) into the escapement support plate (4) as far as possible. Push the escape wheel (5) onto the shaft so that the wheel sits against the plate. Ensure the wheel is attached in the orientation shown. Use Loctite to secure the wheel to the shaft.
2	Position the pallet (6) 1 mm from the end of the pallet shaft (20), and secure with Loctite. Insert the pallet shaft into the escapement support plate in the orientation shown.
3	Push the escapement support plate and the actuator support plate (7) together.
4	Attach the pallet lever arm (8) to the pallet shaft so the lever sits 1 mm from the actuator support plate. Secure the connection with Loctite.

##### 5.5.5 Electronics sub-assembly

**Fig. 8:**
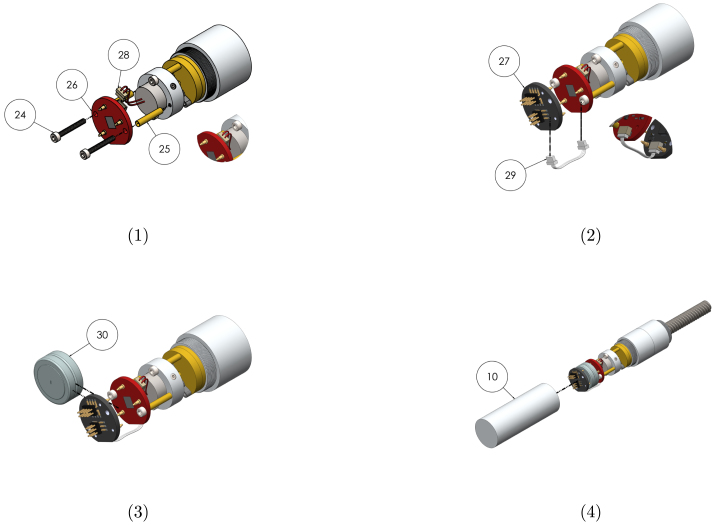
Graphical build and integration instructions for electronics sub-assembly.

Graphical instructions for assembling and integrating the electronics sub-assembly (sub-assembly F as identified in Fig. 2) are provided in Fig. 8. Each assembly step is detailed in Table 13. Components are numbered with reference to Table 2, Table 3.

**Table 12:** Assembly and integration instructions for the actuator sub-assembly. Component numbering per Tables 2–3.

Step	Instruction
1	Press the motor mount (9) onto the shutter motor (21) so that the mount sits against the flange of the motor. Insert grub screws (35) into the threaded holes, and tighten onto the motor using a 1.5 mm hex key.
2	Insert M1.6 × 16 mm socket screws (22) into the clearance holes of the mount, and place a 5.5 mm spacer (23) onto each of the screws.
3	Insert the socket screws into the clearance holes of the escapement sub-assembly. Ensure the pin of the motor arm sits inside the slot of the pallet lever arm (see detail view). The motor may need to rotated in the mount for proper alignment.
4	To integrate the actuator and escapement with the gearbox and leadscrew assemblies, insert the socket screws into the threaded holes of the gearbox housing and tighten using a 1.5 mm hex key.

##### 5.5.6 Reservoir and plunger sub-assembly

**Fig. 9:**
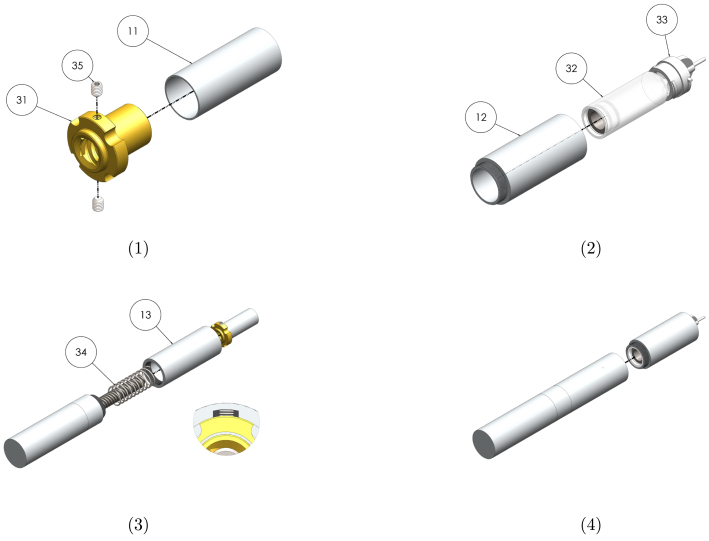
Graphical build and integration instructions for the reservoir and plunger sub-assembly.

Graphical instructions for assembling and integrating the reservoir and plunger sub-assembly (sub-assembly A as identified in Fig. 2) are provided in Fig. 9. Each assembly step is detailed in Table 14. Components are numbered with reference to Table 2, Table 3.

**Table 13:** Assembly and integration instructions for the electronics sub-assembly. Component numbering per Tables 2–3.

Step	Instruction
1	Insert the M1.6 × 14 mm socket screws (24) through the B1 board (26) so that the head of the screws sits on the battery contact side of the board. Place a 10 mm spacer (25) onto each of the screws. Insert the screws into the threaded holes of the actuator motor mount and tighten using a 1.5 mm hex key.
2	Insert the motor cable into the two-pin connector of B1 (26). Connect B2 (27) to B1 using the 3 pin connector cable. Ensure the battery contacts of B2 face B1.
3	Insert two CR1632 batteries (cylindrical sides wrapped in Kapton tape) between the two boards, with the negative terminal of the battery against the black board.
4	Screw the electronics housing (10) onto the gearbox housing. If the internal shoulder in the electronics housing does not apply gentle pressure to B2 (27), create a spacer to ensure good contact pressure.

**Table 14:** Assembly and integration instructions for the reservoir and plunger sub-assembly. Component numbering per Tables 2–3.

Step	Instruction
1	Secure the plunger (11) to the base of the leadscrew nut (31) using Loctite. Insert M2 grub screws (35) into the threaded holes and tighten using a 1.5 mm hex key until the flat of the grub screw extends 1 mm from the surface of the flange. Secure the grub screws in place using Loctite.
2	Fill the reservoir (32) and connect the infusion set (33) as per the manufacturer’s instructions. Insert the reservoir into the reservoir housing (12) and lock the infusion set to the housing using the threaded fitting.
3	Place the compression spring (34) over the leadscrew. Screw the spring housing (13) onto the leadscrew housing. Thread the plunger onto the leadscrew, ensuring the grub screws sit in the internal slots of the spring housing. Press the plunger down as far as it will go.
4	Screw the reservoir housing onto the spring housing.

### 6 Operation instructions

#### 6.1 Loading and programming procedure

After assembling the pump, the following procedure should be used to program, load, and prime the pump. Software is provided only to configure continuous, un-changing delivery. Behaviour can be adapted to specific needs, including real-time changes, by incorporating Bluetooth communication, or a physical button mounted to the device. SPI and other peripherals are broken out from the rear of B2 (27) to enable significant customization.

Users should be aware the current design is a proof-of-concept prototype, and has not been proven to meet the requirements to act as an in-vivo medical device.

**Fig. 10:**
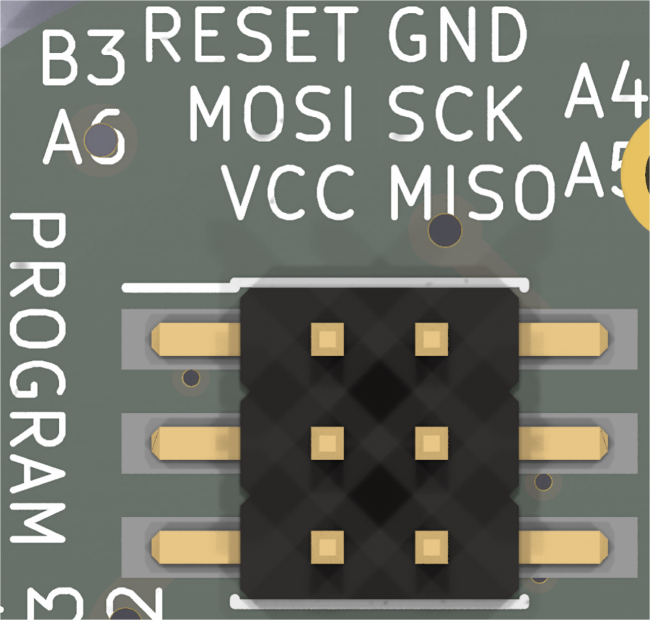
Pinout of the programming header on B2 (27).

1. Input the desired rate of continuous infusion into the code (continuousDelivery.ino).
2. Program the ATTiny441 using a suitable programmer, for example an Arduino Nano configured as an in-system programmer. The pinout for the programming header can be seen in Fig. 10.
3. Load the pump by pushing on the plunger until the spring is fully compressed.
4. Fill the reservoir with the desired fluid using a syringe or vial, so that the rubber plunger of the reservoir sits flush with the end of the reservoir. Connect the infusion set to the reservoir, as per the manufacturer’s instructions.
5. Insert the reservoir into the reservoir housing, as shown in Section 5.5.6.
6. Place the free end of the infusion set into a beaker. Screw the reservoir housing onto the spring housing. The pump plunger will engage with the reservoir plunger as the housing components are connected. Fluid will be flushed through the infusion line into the beaker, priming the infusion line.

#### 6.2 Operational safety hazards

Potential safety hazards during operation of the device along with measures to mitigate the risk are listed in Table 15.

### 7 Validation and characterization

#### 7.1 Accuracy testing to IEC 60601-2-24(2012)

**Fig. 11:**
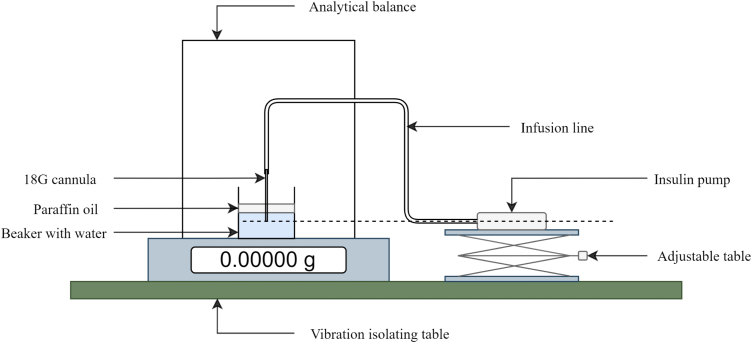
Testing setup to IEC 60601-2-24.

The accuracy of the clockwork pump was assessed according to the test settings and evaluation methods provided in IEC 60601-2-24(2012), an international standard outlining safety and performance requirements for infusion pumps [17]. The IEC compliant testing set-up is shown in Fig. 11. The pump delivers the testing fluid through an infusion set, and a 18 gauge needle into a beaker of water. The change in mass of the beaker during testing is measured using an analytical balance (readability of 0.01 mg), and correlated to the volume delivered by the pump.

The flow-rate and bolus accuracy of the clockwork pump were individually assessed to clauses 201.12.103 and 201.12.105 of the IEC standard. To benchmark the clockwork pump against a commercial syringe pump, a Medtronic 640G insulin pump (Medtronic Inc; Northridge, CA, USA) was tested using the same test set-up and protocol. Specific test parameters and results for the flow-rate and bolus tests are presented in Sections 7.1.1, and 7.1.2 respectively.

**Table 15:** Operational risks, and measures to mitigate risk.

Hazard	Potential cause	Mitigation
Injury from spring decompression	Uncontrolled release of compressed spring and plunger during loading.	Follow assembly instructions, ensure the escapement and one-way bearing lock the screw, and ensure the grub screws are installed securely in the plunger.
Injury from needle	Contact with the exposed needle of the infusion set.	Follow manufacturer’s instructions for handling infusion sets and cover exposed needle with a sheath when the pump is not in use.
Injury from sharp edges on manufactured components	Burrs or rough surfaces on pump housings could cause injury when loading and handling the pump.	De-burr all components after manufacture and ensure there are no exposed sharp edges.

The IEC standard does not provide guidelines for quantifying measurement uncertainty. Therefore, the United States Pharmacopeia methodology was used to estimate both repeatability and accuracy contributions to the measurement uncertainty of the balance [18]. Measurement uncertainty arising from repeatability error can be estimated using the balance repeatability, S, a safety factor of 2, and the minimum weight to be measured, $m_{min}$.

(1) $$U=\frac{2\times S}{m_{min}}=\frac{2\times \text{0.014}\;\text{mg}}{\text{1}\;\text{mg}}=2.8\text{\%}$$

Balance repeatability is defined as the standard deviation of at least 10 repeated measurements of a test weight. The repeatability of the balance used for assessing the clockwork pump was found to be 0.014 mg. The minimum weight to be measured is 1 mg, corresponding to the minimum volume displaced by the pump (0.001 ml).

In addition to repeatability error, accuracy error of the balance was assessed from 15 repeated measurements of a test weight with mass greater than 5% of the balance capacity (repeatability errors dominate near limit of balance capacity) [18]. With a 20 g test weight, the average accuracy error was found to be 3.4 × 10^-3^% of the measured weight, which is negligible. The combined measurement uncertainty arising from the balance is therefore considered to be ≤3% of the minimum measured weight, or ≤±0.03mg.

Aside from measurement uncertainties associated with the balance, experimental factors could introduce error. Potential sources of error are listed in Table 16 along with steps taken to mitigate their impact. With a factor of safety of 2 placed on measurement uncertainty, and sources of experimental error effectively controlled, the uncertainty for each individual measurement is conservatively estimated as ±0.03mg.

**Table 16:** Sources of experimental error and mitigation strategies.

Source	Description and mitigation steps
Change in ambient test conditions	Changes in temperature and humidity in the laboratory used for testing could impact results, including the density of the testing solution (and recorded mass). Temperature and relative humidity were measured every 5 min over a period of 24 h to quantify the fluctuations in ambient conditions over the length of a flow-rate test. The average temperature was measured as 20.4 ° C , with variation <2 ° C . The average relative humidity was 58.3%, with variation <13.5%. As the water used for testing was left to equilibrate to room temperature, volume was calculated using the density of water at 20.4 ° C . The variation on calculated volume due to temperature changes (<2 ° C ) is ≤0.05%, and thus considered negligible.
External vibration	External vibrations could have significant impact on the performance of the balance, by introducing loads which interfere with the weight measurement mechanism. Therefore, the balance was placed on an active vibration isolating table (Vibraplane, Boston, MA, USA).
Eccentric loading of balance	If the load is not centred on the weighing pan, eccentricity error could impact the measurement. To reduce potential eccentricity error, the beaker was centred on the weighing pan. The level of the balance was also monitored throughout testing.
Evaporation	Evaporation of water from the beaker could impact the measured mass, which is of particular concern in flow-rate tests which run for 24 h . To reduce likelihood of evaporation, a 1.5 mm layer of paraffin oil was placed on top of the water in the weighing beaker. The use of paraffin oil is common in studies investigating the accuracy of commercial insulin pumps, and is proven to improve test accuracy [19], [20], [21], [22].
Air in testing solution	Air in the testing solution would impact the measured mass. A testing solution of ISO 3696:1987 class 3 water was used, as per the IEC standard. The water was de-gassed to reduce bubble formation, and limit the resulting variation in mass.
Siphoning effects	Siphoning effects could occur if the pump is not vertically aligned with the cannula tip. As shown in Fig. 11, an adjustable table was used to ensure the pump centre-line was level with the cannula tip.
Blockage in the infusion line	If a blockage developed in the infusion line, the test would be compromised. As per the IEC standard, a new infusion set was used for every flow-rate test. Frequent changes of the infusion line limits the chance of blockages developing.

##### 7.1.1 Flow-rate accuracy

**Fig. 12:**
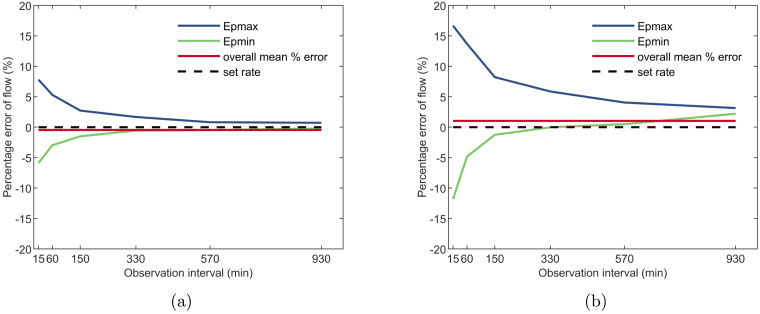
(a) Trumpet curve plot for the commercial insulin pump at a flow-rate of 0.01 ml h^−1^ (b) Trumpet curve plot for the clockwork pump at a flow-rate of 0.01 ml h^−1^.

Flow-rate testing was carried out in accordance with clause 201.12.103 of the IEC standard. An intermediate flow-rate rate was assessed over an analysis period of 24 h, with a sampling interval of 15 min. To enable comparison to a commercial pump (insulin pump), a flow-rate of 0.01 ml h^−1^ was selected, as it is considered an intermediate rate for insulin therapy [20]. The maximum positive and negative percentage errors on flow ($E_{pmax}$ and $E_{pmin}$ respectively) were calculated for six discrete sliding observation windows (15 min, 60 min, 150 min, 330 min, 570 min, and 930 min) and plotted as ‘trumpet’ curves, the standard specified method for presenting flow-rate accuracy data. Trumpet plots for the commercial and clockwork pumps are presented in Fig. 12, Fig. 12, respectively.

The flow-rate is calculated from the total recorded mass in each observation window. The largest relative measurement uncertainty will occur for the 15 min observation window, due to the smallest recorded mass (≈2.5 mg). The relative uncertainty on the measurement for the 15 min window is ±1.2%. For larger observation windows, the recorded mass is greater, and the relative uncertainty decreases. Due to hardware differences between the two pumps, the number of doses each device delivers over the hour to provide a given flow-rate differ. The clockwork pump delivers 10 individual doses (cycle time of 6 min) while the commercial device delivers 20 (cycle time of 3 min). Clause 201.12.103 the standard does not specify a required cycle time (or individual dose size) for any given flow-rate.

The maximum positive and negative flow errors for the commercial device at an observation window of 15 min are 7.8% and −5.8% respectively. The clockwork pump presents greater error for the same observation window duration, with maximum flow errors of 16.7% and −11.8%. As shown in Fig. 12, Fig. 12, error decreases as the observation window widens, which is an expected result due to greater averaging within the window. At the standard based 930 min observation window, the two pumps demonstrate similar error range with 0.7% to −0.2% for the commercial device compared to 3.1% to 2.2% for the clockwork pump. The commercial pump delivers 20 doses per hour compared to 10 for the clockwork pump, so the results are averaged across a greater range of individual doses, which yields smaller error.

$E_{pmax}$ and $E_{pmin}$ remain symmetrical about the set rate for the commercial pump, which indicates similar positive and negative error across the entire test, which averages out to an insignificant overall mean error of −0.5%. The clockwork pump consistently over-delivers throughout the test. As a result, $E_{pmin}$ becomes positive, due to the small systemic bias in the current prototype, as shown in the 930 min observation window, resulting in a larger overall mean error of 1.0%. Overall, accuracy testing of the clockwork pump prototype produced promising results. While the clockwork pump mean error is greater than the commercial pump, further development and manufacture of the design should yield a device with very similar performance. The results validate the novel pumping concept, proving adequate accuracy can be obtained.

**Fig. 13:**
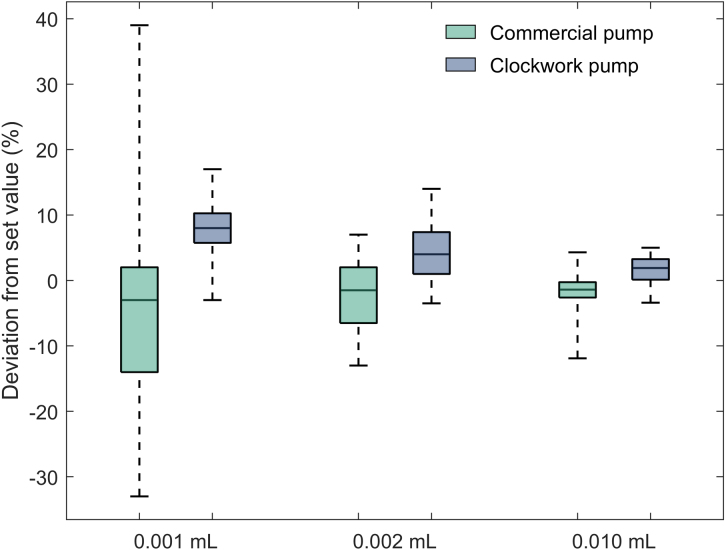
Box plot comparing bolus accuracy of commercial insulin pump (Medtronic 640G) to the clockwork pump.

##### 7.1.2 Bolus accuracy

Bolus testing was conducted to the protocol outlined in clause 201.12.105 of the IEC standard by measuring the mass displaced by 25 successive bolus deliveries for each bolus volume tested. The minimum setting for the pump was tested, which for the clockwork pump is 0.001 ml. Two other volumes were also tested, 0.002 ml, and 0.01 ml. The standard does not specify a settling time between measurements, so a 2 min period was implemented to ensure consistency across experiments. Testing found a 2 min period was adequate to achieve a stable reading. The uncertainty of each measurement is ±0.03mg. For measurements of 0.001 ml, 0.002 ml, and 0.01 ml boluses, the relative uncertainty is ±3%, ±1.5%, and ±0.3% respectively.

Bolus accuracy, presented as relative deviation from the set value (expressed as a percent) for all bolus volumes and pumps tested is shown in boxplots in Fig. 13. The vertical lines on the boxplot indicate the maximum negative deviation and maximum positive deviation from the set value from the series of 25 boluses. The upper and lower limits of the box indicate the upper and lower quartiles of deviation, and the middle line separating the box represents the median deviation. For dose sizes of 0.001 ml and 0.002 ml, the clockwork pump demonstrates improved consistency compared to the commercial device with decreased interquartile ranges (4.5% and 6.4% compared to 16.0% and 8.5%, respectively). The overall range for the commercial device at a volume of 0.001 ml is also significantly greater than the clockwork pump (72% compared to 20%). For a dose size of 0.01 ml, the clockwork pump interquartile range is slightly greater than the commercial device (3.1% compared to 2.9%). However, the overall range is lower (8.4% compared to 16.2%), which indicates similar consistency. The larger relative interquartile range associated with 0.002 ml compared to 0.001 ml for the clockwork pump is an unexpected result, but can likely be attributed to the small sample size (25) specified in the standard. Further testing is required to better quantify pump performance.

Despite exhibiting improved or similar consistency to the commercial device, the median error for the clockwork pump is greater than the commercial device across all three bolus volumes (8.0%, 4.0%, and 1.9% compared to −3.0%, −1.5% and −1.4% for 0.001 ml, 0.002 ml, 0.01 ml volumes respectively). Low variability with a consistent positive median error indicates a systemic error, such as a slightly different gear ratio, or leadscrew pitch than expected. Systematic errors can be eliminated with further development and testing, and/or more precise fabrication.

#### 7.2 Theoretical pump resolution

The linear distance the plunger travels per actuation, $x_{tick}$, can be calculated using the gear ratio, GR, number of teeth on the escape wheel, $t_{n}$, and the lead of the leadscrew, l. The theoretical volume displaced from the reservoir is then found using the reservoir inner diameter, $D_{res}$:

(2) $$x_{tick}=\frac{l}{t_{N}\times GR}=\frac{\text{8}\;\text{mm}}{15\times 60}=\text{8.9}\times \text{10}{}^{\mathrm{-3}}\;\text{mm}$$

(3) $$V_{tick}=\frac{\pi \times D_{res}^{2}}{4}\times x_{tick}=\frac{\pi \times \text{12}\times \text{10}{}^{\mathrm{-3}}\;{\text{m}}^{2}}{4}\times \text{8.9}\times \text{10}{}^{\mathrm{-6}}\;\text{m}=\text{0.001}\;\text{ml}$$

The minimum resolution of the pump can easily be adjusted by altering the leadscrew lead, gear ratio, number of escape wheel teeth, or the diameter of the reservoir. Note for fine leads, the screw becomes self-locking, and cannot be back-driven. Section 7.4 presents the method to estimate the minimum lead required to ensure the screw can be back-driven. Increasing the gear ratio also increases the torque required to rotate the escape wheel, and thus, the spring force required. The number of escape wheel teeth and the reservoir diameter can be changed without significantly altering the spring force and torques. If the reservoir inner diameter was reduced by half to 6 mm, the minimum delivery of the pump would decrease 4× to 2.5 × 10^-4^ ml.

#### 7.3 Torque measurement

**Fig. 14:**
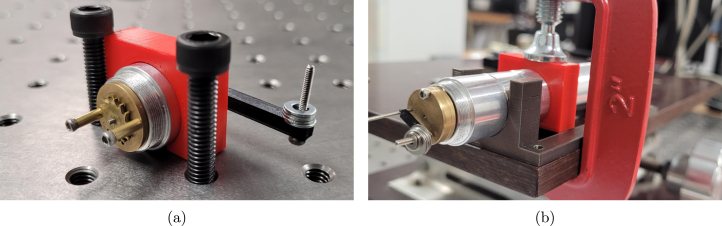
(a) Apparatus for the measurement of escape wheel starting torque (b) Apparatus for the measurement of pallet actuation torque.

The torque to rotate the escape gear through the gearbox, and the torque required to actuate the pallet of the escapement were measured. For both cases, a mass at the end of a lever was used to find the force, and torque required to initiate rotation. The test apparatus for escape wheel rotation and pallet rotation are shown in Fig. 14, Fig. 14, respectively. Washers were used as the mass, and were incrementally added to the end of the 3D-printed lever, until rotation occurred. Using the force ($F_{escape}$ and $F_{pallet}$) and known lever length ($L_{escape}$ and $L_{pallet}$), the torques $T_{escape}$ and $T_{pallet}$ were calculated:

(4) $$T_{escape}=F_{escape}\times L_{escape}=\text{0.018}\;\text{N}\times \text{0.030}\;\text{m}=\text{5.4}\times \text{10}{}^{\mathrm{-4}}\;\text{N}\;\text{m}$$

(5) $$T_{pallet}=F_{pallet}\times L_{pallet}=\text{0.008}\;\text{N}\times \text{0.010}\;\text{m}=\text{8.0}\times \text{10}{}^{\mathrm{-5}}\;\text{N}\;\text{m}$$

#### 7.4 Spring validation and calculation of forces, torques and pressures

To select a spring suitable for insulin delivery, a minimum required spring stiffness was calculated. The stiffness of various off-the-shelf springs were estimated, and a spring meeting the required stiffness selected. The minimum and maximum plunger force, and achievable pumping pressure, were then calculated along with the maximum generated leadscrew torque. Table 17 lists the variable definitions and values required for all calculations.

**Table 17:** Variable definitions, values, and source for validating spring selection.

Variable	Definition	Value	Source
$d_{m}$	Mean screw diameter	7 × 10^-3^ m	Leadscrew property
θ	Thread angle	15°	Leadscrew property
l	Leadscrew lead	8 × 10^-3^ m	Leadscrew property
$f_{s}$	Starting friction of brass nut	0.30	Tabulated value [23]
$T_{escape}$	Escapement starting torque	5.4 × 10^-4^ N m	Measured (see Section 7.3)
$F_{d}$	Force to deliver 1.0 × 10^-6^ l in 1 s	0.8 N	Estimated (see Appendix)
FOS	Factor of safety	1.5	Design parameter
$x_{min}$	Minimum spring compression	12.0 × 10^-3^ m	Design parameter
$x_{max}$	Maximum spring compression	40.0 × 10^-3^ m	Design parameter
d	Spring wire diameter	1 × 10^-3^ m	Spring property
G	Shear modulus	79.3 × 10^9^ N m^−2^	Tabulated value for steel [24]
D	Mean spring diameter	13 × 10^-3^ m	Spring property
N	Number of active spring coils	9	Spring property
$A_{res}$	Cross-sectional area of reservoir	1.13 × 10^-4^ m^2^	Reservoir property

##### 7.4.1 Required spring stiffness

The selected spring must provide sufficient axial force across the entire working range of the plunger to back-drive the leadscrew and generate adequate pressure to deliver fluid. To ensure the selected spring meets the force requirements for successful operation of the pump, a condition for back-driving involving all forces and torques present in the system must be established. Back-driving will occur when $T_{L}\leq 0$, where $T_{L}$ is the lowering torque [23]. The lowering torque is the torque required to rotate the lead-screw so the plunger is advanced in the same direction as the load (spring force). A negative $T_{L}$ indicates the load will advance the plunger, and drive fluid from the reservoir through rotation of the screw without any external effort (back-driving).

**Fig. 15:**
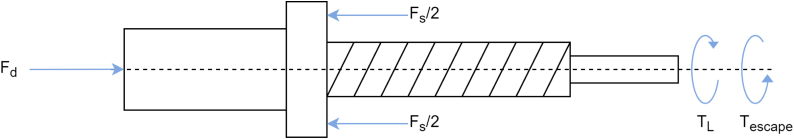
Free body diagram of leadscrew and plunger.

As shown in Fig. 15, the spring force driving the plunger, $F_{s}$, is opposed by the delivery force, $F_{d}$. Rotation of the leadscrew is also opposed by the starting torque of the escape wheel, $T_{escape}$. Note, the delivery force depends on the pumping application. A value of $F_{d}$ appropriate for initial testing and development of the device as an insulin pump was estimated, with details provided in Appendix. To adapt the design for another application, $F_{d}$ should be measured, or calculated from a minimum or maximum pressure requirement.

An equation for $T_{L}$ in terms of $F_{s}$, $F_{d}$ and $T_{escape}$ can be formed:

(6) $$T_{L}=\frac{d_{m}\left(F_{s}-F_{d}\right)}{2}\cdot \frac{\pi f_{s}d_{m}-lcos\left(\theta \right)}{\pi d_{m}cos\left(\theta \right)+f_{s}l}+T_{escape}\;\left(\text{N}\;\text{m}\right)$$

Where all variable definitions, values, and sources are listed in Table 17. The minimum spring force required for back-driving is found by rearranging Eq. (6) and setting $T_{L}=0$:

(7) $$F_{s\left(min\right)}=\frac{-2T_{escape}}{dm}\cdot \frac{\pi d_{m}cos\left(\theta \right)+f_{s}l}{\pi f_{s}d_{m}-lcos\left(\theta \right)}+F_{d}=\text{3.9}\;\text{N}$$

The spring stiffness required to ensure adequate spring force is provided across the entire working range of the spring, $K_{req}$, can be found using Hooke’s law:

(8) $$k_{req}=\frac{F_{s\left(min\right)}\times FOS}{x_{min}}=\text{490}\;\text{N}\;\text{m}{}^{\mathrm{-1}}$$

##### 7.4.2 Validation of selected spring

To find a suitable spring, a variety of compression springs 50 mm in length with varying wire diameters, d, and mean spring diameters, D, were found. The spring stiffness for each spring was estimated using a strain energy approach, and the spring which most closely matched the required stiffness identified [25]. Estimation of the spring stiffness for the closest matching spring is presented:

(9) $$k_{s}=\frac{d^{4}G}{8D^{3}N}=\text{500}\;\text{N}\;\text{m}{}^{\mathrm{-1}}$$

As $K_{s}\geq K_{req}$, the spring will provide adequate force across the entire working range of the plunger to back-drive the leadscrew and ensure all fluid in the reservoir can be delivered.

##### 7.4.3 Minimum and maximum spring forces and pumping pressure

The minimum and maximum axial forces acting on the plunger, $F_{min}$ and $F_{max}$, can be calculated using the minimum and maximum spring compression of the chosen spring:

(10) $$F_{min}=k_{s}\times x_{min}=\text{500}\;\text{N}\;\text{m}{}^{\mathrm{-1}}\times \text{12}\times \text{10}{}^{\mathrm{-3}}\;\text{m}=\text{6}\;\text{N}$$

(11) $$F_{max}=k_{s}\times x_{max}=\text{500}\;\text{N}\;\text{m}{}^{\mathrm{-1}}\times \text{40}\times \text{10}{}^{\mathrm{-3}}\;\text{m}=\text{20}\;\text{N}$$

The minimum and maximum pressure applied to the fluid in the reservoir by the plunger can then be calculated using $F_{min}$ and $F_{max}$, and the internal cross-sectional area of the fluid reservoir.

(12) $$P_{min}=\frac{F_{min}}{A_{res}}\approx \text{50}\;\text{kPa}$$

(13) $$P_{max}=\frac{F_{max}}{A_{res}}\approx \text{175}\;\text{kPa}$$

##### 7.4.4 Maximum leadscrew torque

Maximum torque experienced by the leadscrew, $T_{max}$, must be known to assess the suitability of the one-way bearing. $F_{max}$ can be substituted for $F_{s}$ in Eq. (6) to find a maximum leadscrew torque of 2.8 × 10^-3^ N m.

#### 7.5 Battery-life estimate

To estimate pump battery life, use as an insulin pump is considered, where a delivery requirement of up to 1 ml per day can be expected. All variables, values, and sources of values required for the battery life estimate are listed in Table 18. The average electromagnet current required to deliver 1 ml per day, $I_{motor}$, can be calculated:

(14) $$I_{motor}=\frac{t_{actuation}N_{actuation}}{t_{d}}\cdot I_{coil}=\text{5.6}\times \text{10}{}^{\mathrm{-3}}\;\text{mA}$$

The average on-board processor current required to deliver the required daily dose, $I_{processor}$, can be calculated:

(15) $$I_{processor}=\frac{t_{a}I_{a}+t_{i}I_{i}+t_{s}I_{s}}{t_{d}}=\text{1.4}\times \text{10}{}^{\mathrm{-3}}\;\text{mA}$$

The pump battery life, $T_{battery}$, can be then be estimated:

(16) $$T_{battery}=\frac{C_{battery}}{FOS*\left(I_{motor}+I_{processor}\right)}=\text{9.3}\times \text{10}{}^{3}\;\text{h}\approx 1\mathrm{year}$$

FOS=2 was used as the peak current is greater than the peak pulse current stated within the battery datasheet [27].

**Table 18:** Variable definitions, values, and source for battery life estimate.

Variable	Definition	Value	Source
$t_{actuation}$	Electromagnetic actuation time	40 ms	Design parameter
$N_{actuation}$	Number of actuations per day	1000	Design parameter
$t_{d}$	Time in day	86.4 × 10^6^ ms	–
$I_{coil}$	Electromagnet coil current	12.2 mA	Measured steady-state current
$t_{a}$	Processor active time	24 000 ms	Design parameter
$I_{a}$	Processor active current	0.2 mA	Product data-sheet [26]
$t_{i}$	Processor idle time	24 700 ms	Design parameter
$I_{i}$	Processor idle current	0.03 mA	Product data-sheet [26]
$t_{s}$	Processor sleep time	86.35 × 10^6^ ms	Design parameter
$I_{s}$	Processor sleep current	0.0013 mA	Product data-sheet [26]
$C_{battery}$	Battery capacity	130 mA h	Product data-sheet [27]
FOS	Factor of safety	2	Selected parameter

#### 7.6 Failure considerations

##### 7.6.1 Circlip failure

**Fig. 16:**
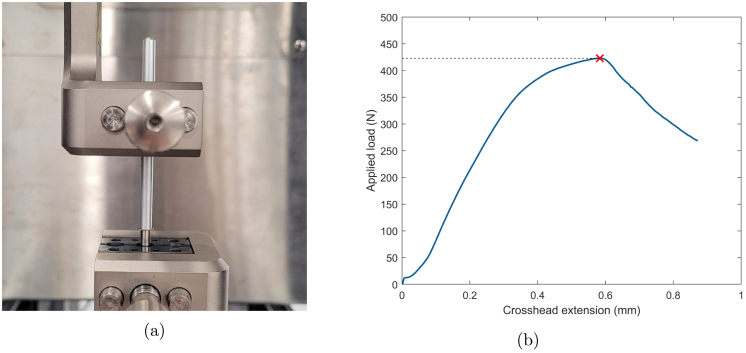
(a) Apparatus for circlip testing (b) Load cell readout with circlip failure load indicated.

The circlip transfers the axial spring load to the thrust bearing. To assess the load bearing capacity of the circlip, an MTS Criterion (MTS Systems Corporation, MN, USA) tensometer was used to apply an axial load directly onto a circlip sitting in the groove of a test shaft. As shown in Fig. 16(a), the test shaft was attached to the bottom jaw of the tensometer. A 4.05 mm ID aluminium tube was attached to the top jaw so the load could be applied directly to the circlip. The load was applied with a set crosshead movement of 1 mm min^−1^.

The load cell readout is presented in Fig. 16(b), and shows a peak load of 423 N, before failure (indicated by a reduction in applied load). The factor of safety on circlip failure is calculated using the circlip failure load, $F_{failure}$, and the maximum spring force:

(17) $$FOS_{circlip}=\frac{F_{failure}}{F_{max}}=\frac{423}{20}=21.2$$

##### 7.6.2 One-way bearing failure

The factor of safety on one-way bearing failure is calculated by comparing the maximum leadscrew torque to the bearing manufacturer’s published torque limit. The torque limit (0.34 N m) represents the maximum torque that can be applied to the bearing without slippage, for a lifetime of 1 million locking cycles, which occur when reloading the pump [28].

(18) $$FOS_{bearing}=\frac{T_{limit}}{T_{max}}\approx 120$$

##### 7.6.3 Gear failure

The tangential load applied to the input gear of the gearbox by the leadscrew, $W_{t}$, can be estimated using the maximum torque, $T_{max}$, and the pitch diameter of the gear, $d_{gear}$:

(19) $$W_{t}=\frac{2\times T_{max}}{d_{gear}}$$

It is suggested the approach for estimating gear contact and bending stress outlined in the ANSI/AGMA 2001-D04 standard is followed [29]. The standard provides methodology for rating the pitting resistance and bending strength of spur and helical involute gear pairs, and accounts for fatigue loading. Due to the complexity of the calculations and unknown characteristics of the current gearbox, including material hardness and surface condition, the analysis is not presented here. If the design is further developed for applications where gearbox failure would have severe consequences, it is essential a fatigue-based gear analysis is conducted to ensure the selected gearbox can safely support the load for the pump lifetime.

#### 7.7 Design limitations

Limitations of the clockwork pump design are identified in Table 19, along with potential implications.

**Table 19:** Design limitations and implications.

Limitation	Description and implication
No fail-safe mechanism	If the escapement is decoupled from the leadscrew, there is currently no mechanism to prevent the delivery of all fluid in the reservoir. The escapement could become decoupled if the one-way bearing fails, the gearbox shaft slips in the gearbox connector, the gearbox fails, or the escape wheel slips on its shaft.
Excess spring force	As the compression spring is selected to provide sufficient force to back-drive the leadscrew when the plunger is at the minimum spring compression position, there is excess spring force at all other plunger positions. Extra spring force places greater load on key components such as the gearbox, increasing wear and reducing pump life.
Variable pumping pressure	Achievable pressure decreases from ≈175 kPa at maximum spring compression to ≈50 kPa at minimum spring compression. The large pressure drop is a limitation as if the required pressure exceeds the achievable pressure at any stage of delivery, the pump will fail to deliver. The pump will also not be suitable for a situation which must provide a constant pressure throughout pump operation.
Unsupported axial load	Only the axial load in the pumping direction is supported by the thrust bearing. The axial force on the leadscrew when loading the pump is currently supported by the gearbox connector and gearbox, which places unnecessary load on these components.
Limited pumping resolution	Although the pumping resolution of the design can be modified easily, it is unlikely that the resolution achieved by motor driven pumps with high gearing can be matched. For the majority of applications, the resolution achievable with the clockwork pump will be adequate.

### 8 Conclusion

A design for low-cost, low-power, portable, and programmable syringe pump is presented. The device is highly customizable, but is intended for eventual use as an insulin pump. The pump design is made accessible through construction from off-the-shelf or readily manufactured components. The flow-rate and bolus delivery accuracy of the device are similar to a commercial insulin pump. The testing results validate the novel pumping mechanism which utilizes a clockwork mechanism to achieve low-power delivery of fluid from the reservoir, showing it can compete with motor-driven devices. A systematic error in the current prototype was identified, with the pump consistently over-delivering. Further development of the design and incorporation of higher precision hardware will likely rectify the issue. Bluetooth control of the device will be included in future design iterations, allowing the user to alter flow-rates, and select bolus doses real-time, using a mobile phone.

### Human and animal rights

No human or animal studies were conducted in the design of this work.

## Declaration of competing interest

None.

## Figures and Tables

**Table 20:** Variable definitions, values, and source for estimating delivery force.

Variable	Definition	Value	Source
$V_{dose}$	Dose volume	1.0 × 10^-9^ m^3^	Reservoir property
$A_{inf}$	Cross-sectional area of infusion line	1.12 × 10^-7^ m^2^	Infusion line property
$t_{dose}$	Time to deliver dose	1 s	Selected time period
$\rho_{water}$	Density of water	997 kg m^−3^	Reference value
g	Acceleration due to gravity	9.81 m s^−2^	Reference value
h	Height difference	0.7 m	Length of infusion line
$A_{res}$	Cross-sectional area of reservoir	1.13 × 10^-4^ m^2^	Infusion line property

## Appendix

To obtain a realistic estimate of the delivery force, $F_{d}$, during operation of the device as an insulin pump, a simple energy balance was performed. A requirement for the pump to deliver 0.001 ml over a period of 1 s was established. While arbitrary, the dose size and delivery time-frame were deemed reasonable for initial development of a pump targeting insulin therapy. As the infusion line is 0.7 m long, a height difference between the pump and the delivery point of 0.7 m was selected, to represent the situation requiring greatest pumping pressure. The variables, values, and value sources used for all calculations are included in Table 20.

The linear distance each 0.001 ml dose travels through the infusion line, and fluid velocity required to deliver the dose over a period of 1 s can be calculated:

(20) $$d_{dose}=\frac{V_{dose}}{A_{inf}}=\text{0.009}\;\text{m}$$

(21) $$v_{dose}=\frac{d_{dose}}{t_{dose}}=\text{0.009}\;\text{m}\;\text{s}{}^{\mathrm{-1}}$$

The delivery force, $F_{d}$ can then be estimated using a simple energy balance:

(22) $$F_{d}=A_{res}\cdot \left(\rho_{water}\cdot \left(\frac{1}{2}v_{dose}^{2}+gh\right)\right)=\text{0.8}\;\text{N}$$
